# Electron transfer through arsenite oxidase: Insights into Rieske interaction with cytochrome *c*

**DOI:** 10.1016/j.bbabio.2017.08.003

**Published:** 2017-10

**Authors:** Cameron Watson, Dimitri Niks, Russ Hille, Marta Vieira, Barbara Schoepp-Cothenet, Alexandra T. Marques, Maria João Romão, Teresa Santos-Silva, Joanne M. Santini

**Affiliations:** a Institute of Structural and Molecular Biology, Division of Biosciences, University College London, WC1E 6BT, United Kingdom; b Department of Biochemistry, University of California; Riverside, Riverside, CA 92521, USA; c UCIBIO-Requimte, Department of Chemistry, Faculty of Sciences and Technology, Universidade Nova de Lisboa, Portugal; d Aix-Marseille Univ, CNRS, BIP UMR 7281, 31 chemin J. Aiguier, 13402 Marseille Cedex 20, France

**Keywords:** Arsenite oxidase, Rate-limiting step, Molybdenum enzyme, Rieske protein, Cytochrome *c*, Stopped-flow spectroscopy, Isothermal titration calorimetry

## Abstract

Arsenic is a widely distributed environmental toxin whose presence in drinking water poses a threat to > 140 million people worldwide. The respiratory enzyme arsenite oxidase from various bacteria catalyses the oxidation of arsenite to arsenate and is being developed as a biosensor for arsenite. The arsenite oxidase from *Rhizobium* sp. str. NT-26 (a member of the Alphaproteobacteria) is a heterotetramer consisting of a large catalytic subunit (AioA), which contains a molybdenum centre and a 3Fe-4S cluster, and a small subunit (AioB) containing a Rieske 2Fe-2S cluster. Stopped-flow spectroscopy and isothermal titration calorimetry (ITC) have been used to better understand electron transfer through the redox-active centres of the enzyme, which is essential for biosensor development. Results show that oxidation of arsenite at the active site is extremely fast with a rate of > 4000 s^− 1^ and reduction of the electron acceptor is rate-limiting. An AioB-F108A mutation results in increased activity with the artificial electron acceptor DCPIP and decreased activity with cytochrome *c*, which in the latter as demonstrated by ITC is not due to an effect on the protein-protein interaction but instead to an effect on electron transfer. These results provide further support that the AioB F108 is important in electron transfer between the Rieske subunit and cytochrome *c* and its absence in the arsenite oxidases from the Betaproteobacteria may explain the inability of these enzymes to use this electron acceptor.

## Highlights

• Reduction of cytochrome *c* is the rate-limiting step of Aio catalysis.
• The reduction of the molybdenum centre by arsenite is very fast.
• The reduction of the Fe-S clusters appears to be simultaneous and reflects the highly cooperative two-electron transfer.
• The AioB F108 is important for electron transfer to cytochrome *c*.

## Graphical abstract

**Figure:**
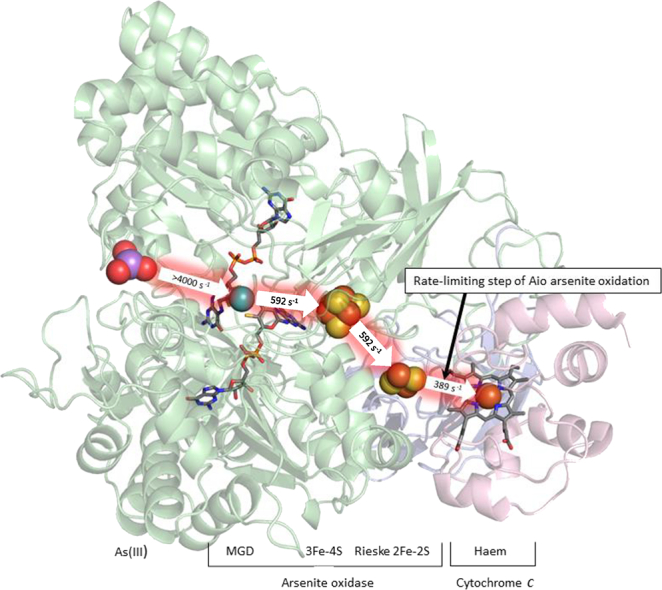

## 1 Introduction

Arsenic, in the inorganic forms arsenite (+ III) and arsenate (+ V), is toxic to most organisms [1]. The reduction potential of the arsenite/arsenate couple (+ 60 mV) [1] is such that certain phylogenetically diverse bacteria can either use arsenite as an electron donor or arsenate as a terminal electron acceptor for growth [2]. Aerobic arsenite oxidation is catalysed by arsenite oxidase, Aio, which couples the oxidation of arsenite to the reduction of oxygen to water generating ATP and in some cases NADH for carbon dioxide fixation [3]. The physiological electron acceptor for Aio has been shown to be *c*-type cytochromes or azurin [4], [5], [6], [7].

**Fig. 1:**
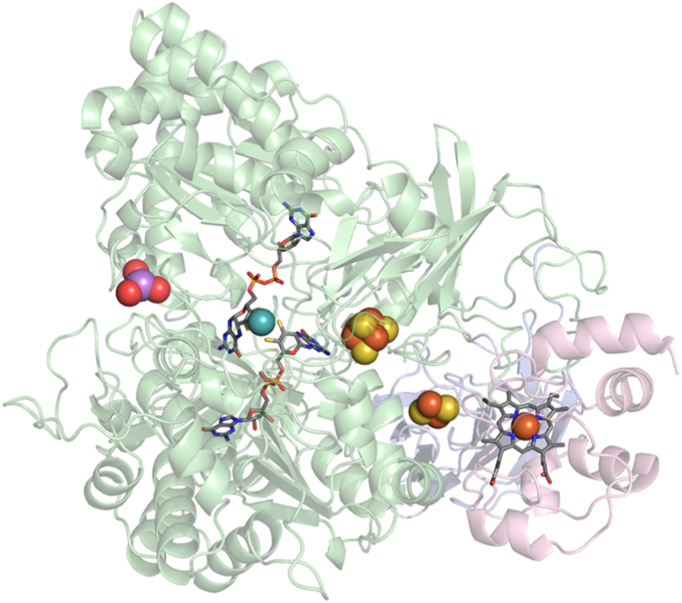
Structure of Aio (AioA, green and AioB, blue) with arsenite (purple) and cytochrome *c* (pink). The molybdenum centre is shown as a blue ball (Mo) and sticks (MGD). The [Fe-S] clusters are shown in yellow and orange. PDB ID: 4AAY (NT-26 Aio) and 1HRC (horse-heart cytochrome *c*).

Aio is a member of the dimethyl sulfoxide reductase (DMSOR) superfamily of molybdoenzymes, which all contain two equivalents of an organic pyranopterin cofactor coordinated to the molybdenum, usually present as the dinucleotide of guanine and termed MGD (for molybdopterin guanine dinucleotide). Aio is unique among members of the DMSOR superfamily, however, in that the molybdenum is not coordinated to the protein by an amino acid side chain [8]. The molybdenum centre of Aio also exhibits highly cooperative two electron transfer, with the intermediate Mo(V) oxidation state not typically observed upon reduction of Mo(VI) to Mo(IV) [9] until recently in a mutant with altered hydrogen bonding to the MGD [10]. Aio also contains a high potential 3Fe-4S cluster in the large catalytic subunit, AioA, rather than the more common 4Fe-4S cluster, and a Rieske centre in the small subunit, AioB, homologous to the Rieske protein in the *bc*_1_ and *b*_6_*f* complexes [11], [12]. Aio is the only molybdoenzyme to contain a Rieske 2Fe-2S cluster (in which one of the Fe atoms is complexed by two histidine residues instead of two cysteines) [13]. An overview of the redox-active centres in Aio catalysis is shown in Fig. 1. It has been suggested that the electrons from arsenite oxidation pass to the molybdenum centre, to the 3Fe-4S cluster, the Rieske cluster and finally to an electron acceptor [8], [13].

Heterologous expression of the NT-26 Aio in *Escherichia coli* has facilitated a more detailed study of the mechanisms of electron transfer [7], [8], [10], [14] and its development as a biosensor for arsenite [15]. In this study, we use a combination of steady-state and stopped-flow kinetics as well as isothermal titration calorimetry (ITC) to determine the rate-limiting step of catalysis. Crystallization and structure determination of the AioB F108A mutant provides evidence of protein integrity and details of the atomic model of the enzyme. Understanding electron transfer through the enzyme is critical to further biosensor development.

## 2 Experimental procedures

### 2.1 Heterologous Aio expression, purification and site-directed mutagenesis

The NT-26 *aioBA*-pPROEX-HTb + construct and *E. coli* strain DH5α were used for the aerobic expression of Aio where the final culture volume was 2 L in a 5 L flask, induction with isopropyl β-D-1-thiogalactopyranoside was at 21 °C for 24 h and the enzyme purified as described previously [8]. Site-directed mutagenesis to obtain the AioB-F108A mutant was done as described previously [8] with the following primers, AioBF108A forward 5′ GTCCTCACAAGGGTGCTCCTCTGAGCTACTCCGC 3′ and AioBF108A reverse 5′ GCGGAGTAGCTCAGAGGAGCACCCTTGTGAGGAC 3′.

### 2.2 Enzyme assays

Aio assays measuring steady-state kinetic parameters for arsenite were performed as described previously using dichlorophenolindophenol (DCPIP) [6] (the ε for DCPIP at pH 5.5 used was 8.2 mM^− 1^ cm^− 1^ [16] instead of 23 mM^− 1^ cm^− 1^ [6]) and horse heart cytochrome *c* (Sigma-Aldrich) [14] as electron acceptors. The steady-state kinetic parameters of cytochrome *c* were determined using an excess of arsenite (2.5 mM) and followed the reduction of cytochrome *c* at 416 nm (Δɛ = 57.5 mM^− 1^ cm^− 1^; based on UV–visible spectra in 50 mM Tris-HCl (pH 8) using ɛ_550_ = 8.4 and 29.5 mM^− 1^ cm^− 1^ for oxidised and reduced cytochrome *c* to determine the concentration [17])_._ Kinetics experiments were performed on three separate occasions with three separate enzyme preparations. NaCl was not added to any buffers as 100 mM reduced the activity of Aio with cytochrome *c* to 35% (data not shown). Using salt is also not physiologically relevant as the Aio is a periplasmic enzyme which means that the pH and salt concentration would be in equilibrium with the environment and NT-26 was isolated from a low-salt environment [18].

### 2.3 Stopped-flow UV–visible spectroscopy

All experiments were conducted in 50 mM Tris-HCl (pH 8) at 5 °C using a SX-20 stopped-flow spectrophotometer (Applied Photophysics, Inc.) equipped with photodiode array and photomultiplier tube detection and running ProData SX 2.2.5.6 acquisition software. A volume of 1.2 mL of 30 μM Aio was placed in a glass tonometer and made anaerobic by stirring in an O_2_ scrubbed Argon rich environment for 1 h. Aio was reacted with 500 μM arsenite in a stopped-flow apparatus to follow the reductive half-reaction. Reduction of the molybdenum centre and [Fe-S] reduction was followed at 680 nm and 450 nm, respectively. To observe the interaction with cytochrome *c*, 30 μM Aio was mixed with 60 μM cytochrome *c* and 30 μM arsenite to catalyse a single turnover. The rate of cytochrome *c* reduction was followed at 551 nm. Four to six replicates were performed with one enzyme preparation. Rate constants thus determined are presented at 25 °C based on the Arrhenius equation [19].

### 2.4 Isothermal titration calorimetry

ITC was carried out using a MicroCal200 ITC and analysed with the accompanying software (GE Healthcare). A volume of 350 μL of 75 μM reduced Aio was loaded into the cell in 50 mM Tris-HCl (pH 8) and 40 μL of 750 μM reduced cytochrome *c* in 50 mM Tris-HCl (pH 8) was loaded into the injection syringe. The cytochrome *c* was injected into the enzyme solution in 1.5 or 2 μL volumes with 1000 rpm stirring at 25 °C. All proteins were reduced prior to the experiment by the addition of excess dithionite which was removed by gel filtration chromatography using either a Superdex 200 (for Aio) or 75 (for cytochrome *c*) gel filtration column (GE Healthcare). ITC experiments were performed on three separate occasions with three independent enzyme preparations.

### 2.5 Statistical analysis

The statistical significance of the differences between WT and AioB-F108A Aio results were determined using a Student's *t*-test.

### 2.6 X-ray crystallography of AioB-F108A mutant

The purified AioB-F108A mutant was subjected to crystallization experiments, based on the previously reported crystallization conditions used for the Aio [8]. Crystals were obtained using the sitting drop vapor diffusion method, where droplets consisting of 2 μL of protein at 12.15 mg/mL and 1 μL of reservoir solution were equilibrated against 500 μL of reservoir solution containing 0.1 M Hepes sodium pH 7–8, 2% PEG 400, 2 M ammonium sulfate. Yellowish crystals grew as thin plates within 3–7 days. For data collection, protein crystals were first transferred into the harvesting solution (0.1 M Hepes sodium pH 7.5, 2% PEG 400, 2.2 M ammonium sulfate), then into cryoprotectant solution (harvesting solution supplemented with 20% glycerol) and finally flash-frozen in liquid nitrogen. Diffraction experiments were carried out at the PXIII beamline at the Swiss Light Source; the crystals diffracted up to a maximum resolution of 2.2 Å (data collection statistics are provided in Table S1). Using X-rays of 1 Å wavelength, a complete data set was collected, processed and scaled using the XDS program package [20]. Data quality was analysed using Aimless from the CCP4 package [21], [22] and structure determination was accomplished by rigid body refinement using Refmac5 [23] with the crystal structure of WT NT-26 Aio as the search model. An anomalous electron density map was generated using CAD and FFT [24]. Interactive cycles of model building and refinement were performed with COOT [25] and Refmac5 [23] software (refinement statistics are shown in Table S1). The atomic coordinates of the AioB-F108A mutant have been deposited in the PDB ID:5NQD.

### 2.7 EPR titration of AioB-F108A mutant

EPR spectroscopy was performed on WT and F108A variant enzymes obtained after the desalting step. Redox titrations were performed at 15 °C, pH 7, as described by Dutton [26] and adapted as described by Duval [27] in the presence of the following redox mediators at 100 mM: 1,4 *p*-benzoquinone, DCPIP, 2,5-dimethyl-*p*-benzoquinone, 2-hydroxy 1,2-naphthoquinone, 1,4-naphthoquinone. Reductive titrations were carried out using sodium dithionite, and oxidative titrations were carried out using ferricyanide. EPR spectra were recorded on a Bruker ElexSys X-band spectrometer fitted with an Oxford Instruments liquid-Helium cryostat and temperature control system. Evaluation were performed on the g = 1.88 signal.

## 3 Results and discussion

### 3.1 The reductive half-reaction of Aio

There are four electron transfer events in the proposed electron pathway for arsenite oxidation catalysed by Aio: arsenite to the molybdenum centre, from the molybdenum centre to the 3Fe-4S cluster, from the 3Fe-4S cluster to the Rieske 2Fe-2S cluster and finally from the Rieske 2Fe-2S cluster to the terminal electron acceptor [13]. Because the molybdenum centre and the Fe-S clusters have distinct spectral signatures at 680 nm and 450 nm, respectively [6], [10], their reduction in the course of the reaction of Aio with arsenite could be followed independently by stopped-flow UV–visible spectroscopy.

**Fig. 2:**
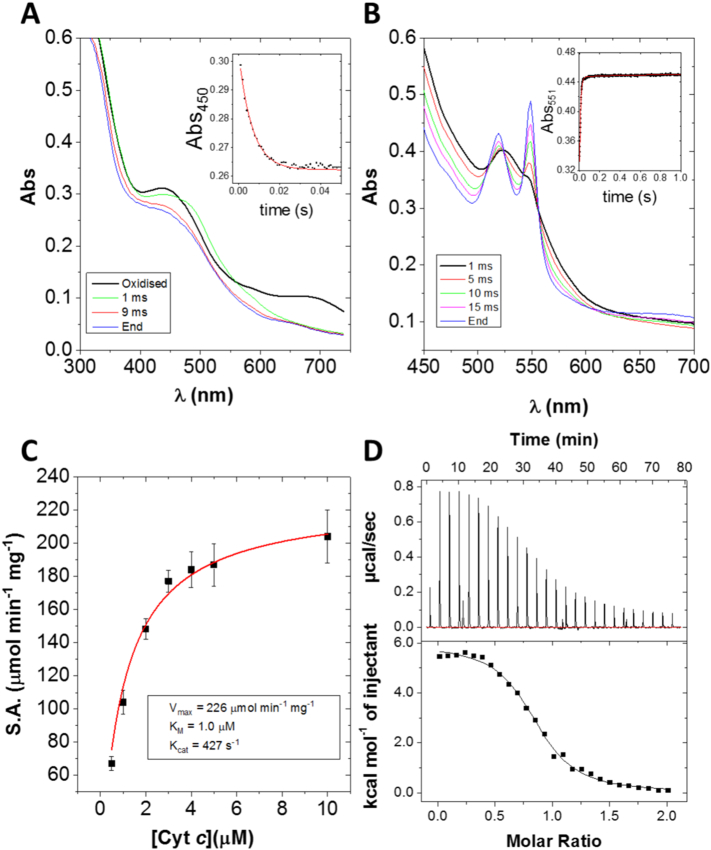
Results of measurements of kinetic and thermodynamic parameters of WT Aio. A) Reductive half-reaction of Aio with arsenite. *Inset*: change in absorbance at 450 nm with single exponential fit (red line). B) Reduction of cytochrome *c* by Aio and arsenite. *Inset*: change in absorbance at 551 nm with double exponential fit (red line). Stopped-flow results are the average of 4–6 repeats with one enzyme preparation. C) Steady-state kinetics of cytochrome *c* and Aio with excess arsenite fit with the Michaelis-Menten model (red line). Results are the average of three separate enzyme preparations. S.A. stands for specific activity. D) *Upper panel*: ITC raw thermogram of Aio titrated against cytochrome *c*. *Lower panel*: Heats integrated with respect to time and plotted against molar ratio with a 1:1 binding fit (black line). Results are the average of three separate enzyme preparations.

In a stopped-flow experiment with Aio, reaction with arsenite results in the immediate quenching of the 680 nm absorption band within the mixing dead-time (~ 1 ms) of the instrument, which implies that the molybdenum centre was reduced by arsenite at a rate of > 4000 s^− 1^. The quenching of the 450 nm band occurred at an arsenite-independent rate of 592 ± 20 s^− 1^ and was monophasic, suggesting that electron transfer rates from the Mo site to the proximal Fe-S and from the proximal to the distal Fe-S are similar and indistinguishable *via* the exponential fitting procedure or alternatively the measured rate represents the ET to the distal Fe-S while the ET from the proximal to the distal centre is considerably faster. Considering that the rates of ET from the Mo centre are typically limited by product release, the latter possibility seems more likely (Fig. 2A).

The very rapid reduction of the molybdenum centre of Aio is at the high end of rate constants observed for the reduction of molybdenum centres for molybdoenzymes, which are quite variable. The reductive half-reaction of DMSO reductase from *Rhodobacter capsulatus*, however, also occurs within the mixing dead time at 10 °C [28]. The molybdenum-containing formate dehydrogenase from *Ralstonia eutropha*, another member of the DMSOR family, is also quite rapid with a rate of 140 s^− 1^ at 10 °C (approximately 400 s^− 1^ at 25 °C) [29]. Chicken liver sulphite oxidase reacts slightly slower with sulfite, with a rate constant of 150–200 s^− 1^ at 25 °C [30]. Some of the slower rates observed are seen with members of the xanthine oxidase family (bovine milk xanthine oxidase reacts with xanthine at 14 s^− 1^ at 25 °C, and *Oligotropha carboxydovorans* CO dehydrogenase reacts with CO at 11.4 s^− 1^ at 4 °C) [31], [32]. Aio is unique among molybdoenzymes in that the Mo(V) state is unstable and unobservable in the WT enzyme [9], [10], and this may be important in rate acceleration at the active site. However, since the reduction of the molybdenum centre occurs in the mixing dead-time for both Aio and the *R. capsulatus* DMSO reductase (which has a stable Mo(V) state [33]), it is not possible to compare their rates to conclude that cooperative electron transfer is important in rapid reaction rates.

The reduction potentials of the molybdenum centre, [3Fe-4S] and Rieske cofactors are similar at + 240 mV, + 270 mV and + 225 mV, respectively [8], [10], [34]. It is unlikely that differences between the cofactor reduction potentials plays a large role in defining electron transfer rates. The rate of [Fe-S] cluster reduction is most likely limited by dissociation of the arsenate product from the Mo active site.

The Fe-S clusters of Aio appear to become reduced simultaneously, consistent with the apparent two-electron re-oxidation of the reduced Mo(IV) centre and failure to observe the Mo(V) state. In most molybdoenzymes, the Mo(V) is sufficiently stable to allow the molybdenum centre to act as a transducer between obligate one- and two-electron transfer centres [35]. The hydrogen-bonding network of the MGD cofactor in Aio appears to have been highly optimised to destabilise the Mo(V) state [10], perhaps due to the instability of As(IV), which rapidly decays to As(V) and superoxide [36]. This may have provided evolutionary selection pressure to eliminate one-electron transfer between arsenite and the molybdenum centre. This being the case, the presence of two Fe-S clusters would be required to permit immediate re-oxidation of the molybdenum centre, allowing the enzyme to act as a transducer and couple the oxidation of arsenite to the reduction of a physiological electron acceptor that can only accept one electron at a time.

### 3.2 The rate of cytochrome *c* reduction by Aio

The final step in arsenite oxidation is electron transfer from the Rieske 2Fe-2S cluster to the terminal electron acceptor [13]. NT-26 Aio is able to reduce both the native cytochrome *c*_552_ [37] and horse heart cytochrome *c* [14], [37].

The reduction of cytochrome *c* by Aio and arsenite is found to be biphasic (Fig. 2B). The first phase accounts for 97% of the absorbance change and has a rate constant of 389 ± 7 s^− 1^. The second phase has a rate constant of 11 ± 6 s^− 1^ and is probably due to Aio and cytochrome *c* achieving equilibrium (which is likely due to the similarity in the Rieske and heme reduction potentials of + 225 mV and + 250 mV, respectively [8], [38]), and is likely to be catalytically irrelevant. The reduction of cytochrome *c* is the slowest process observed in the stopped-flow experiments, which means that the reduction of cytochrome *c* is the rate-limiting step in overall catalysis.

To further support the conclusion that reduction of cytochrome *c* is rate-limiting, the steady-state kinetics of Aio with cytochrome *c* and excess arsenite were measured using 2.5 mM arsenite and 10, 5, 4, 3, 2, 1 and 0.5 μM cytochrome *c* (Fig. 2C). The k_cat_ was 427 ± 32 s^− 1^, which is very similar to the rate of electron transfer from the Rieske cluster to cytochrome *c* observed in the stopped-flow experiments and in good agreement with previously reported values [14]. The K_M_ for cytochrome *c* was 1.0 ± 0.1 μM, and the K_M_ for arsenite was previously reported to be 9.3 ± 1.5 μM [14].

The interaction between Aio and cytochrome *c* was studied further by ITC and the K_d_ for cytochrome *c* was 2.3 ± 0.7 μM (Fig. 2D and Table 1), which is slightly higher than the K_M_. The similar values for K_d_ and K_m_ for cytochrome *c* suggest that either electron transfer or product release (as opposed to substrate binding) is rate-limiting, which is not uncommon in enzymes that follow Michaelis-Menten kinetics under saturating conditions [39]. It is unlikely that product release is rate-limiting as this would have resulted in k_551_ (the rate of cytochrome *c* reduction observed by stopped-flow) being greater than k_cat_ as the former reflects the rate of binding and electron transfer to cytochrome *c* but not product release. Conversely, the k_cat_/K_M_ ratio was 4 × 10^8^ M^− 1^ s^− 1^ which would suggest that the enzyme is ‘catalytically perfect’, meaning that the reaction with cytochrome *c* is diffusion-limited (and therefore electron transfer would not be rate-limiting) [40]. While strictly speaking both these models are accurate for single substrate enzymes, as the K_M_'s of the substrates of double displacement reactions are often altered by the rate of the other half-reaction [41], we consider it more likely that electron transfer is most likely rate-limiting as ‘catalytic perfection’ is a very rare phenomenon.

**Table 1:** Thermodynamic parameters of the binding of cytochrome *c* with WT Aio and AioB-F108A.

Aio	k_d_ (μM)	ΔH (kcal mol^− 1^)	ΔS (cal mol^− 1^ deg^− 1^)	Stoichiometry
WT	2.3 ± 0.7	6.0 ± 0.1	46.1 ± 1.1	0.85 ± 0.15
AioB-F108A	7.8 ± 1.7	3.0 ± 0.3	33.5 ± 1.1	0.82 ± 0.23

Results are the averages and standard deviations of three experiments with three separate enzyme preparations.

Rieske proteins have highly tuneable reduction potentials as well as high structural plasticity, suggesting that they may be involved in electron transfers where the reduction potential has to be very specific or where high promiscuity is beneficial [42], [43]. An example of the Rieske protein's high tuneability has been demonstrated *via* point mutations that alter the hydrogen bonding network of the *bc*_1_ Rieske cluster which induced changes in the reduction potential as large as 130 mV with subsequent reductions in enzyme activity with cytochrome *c* [44]. Members of the Rieske dioxygenase family tend to exhibit a high degree of substrate promiscuity [45], [46], [47]. Gene knock out studies have also shown that NT-26 is able to metabolise arsenite in the absence of the native cytochrome *c*_552_ suggesting that the NT-26 Aio is able to use alternative electron acceptors *in vivo* [5]. In fact, the ability to use alternative electron acceptors has also been observed with the arsenite oxidases from *Alcaligenes faecalis* [6] and *Ralstonia* sp. 22 [7]. There is also growing support for models of modular electron transport chains with high degrees of plasticity, in which different protein-protein complexes form to respond dynamically to changes in the environment [48], [49], [50]. It is therefore possible that electron transfer from the Rieske cluster is rate-limiting in Aio as the structure has been compromised to work with multiple electron acceptors as opposed to optimised for one.

### 3.3 The effect of the AioB-F108A substitution on Aio activity

**Fig. 3:**
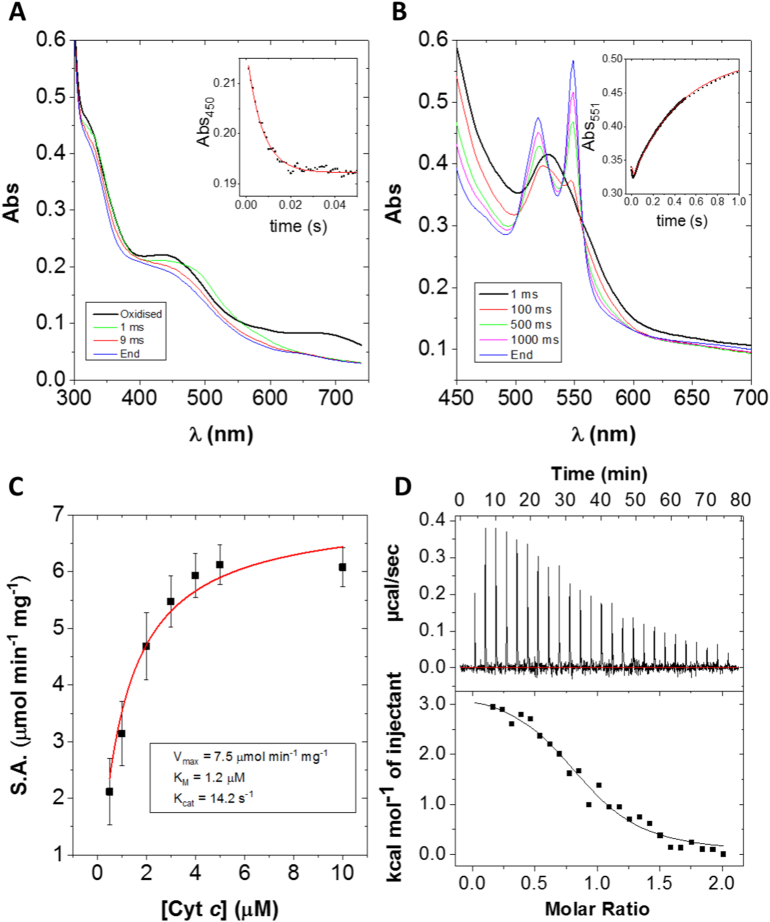
Results of measurements of kinetic and thermodynamic parameters of the AioBF108A mutant. A) Reductive half-reaction of F108A with arsenite. *Inset*: change in absorbance at 450 nm with single exponential fit (red line). B) Reduction of cytochrome *c* by F108A and arsenite. *Inset*: change in absorbance at 551 nm with triple exponential fit (red line). Stopped-flow results are the averages of 4–6 repeats with one enzyme preparation. C) Steady-state kinetics of cytochrome *c* and F108A with excess arsenite fit with the Michaelis-Menten model (red line). Results are the average of three separate enzyme preparations. S.A. stands for specific activity. D) *Upper panel*: ITC raw thermogram of F108A titrated against cytochrome *c*. *Lower panel*: Heats integrated with respect to time and plotted against molar ratio with a 1:1 binding fit (black line). Results are the average of three separate enzyme preparations.

The Rieske subunit of NT-26 AioB lacks a disulphide bridge proximal to the 2Fe-2S cluster, instead containing a phenylalanine and a glycine at the positions otherwise occupied by cysteines in betaproteobacterial arsenite oxidase [8]. Mutation of AioB Phe 108 to Ala (F108A) was made to analyse what effect, if any, removal of the large, hydrophobic and aromatic phenylalanine had on enzyme activity. With cytochrome *c* as an electron acceptor, the k_cat_ was 14.2 ± 0.2 s^− 1^, approximately 30-fold lower than that observed with the WT enzyme (*t*-test p = 0.0001). On the other hand, the K_M_'s for arsenite and cytochrome *c* were 2.0 ± 0.4 μM and 1.2 ± 0.3 μM, respectively (Fig. 3C), essentially unchanged from the WT enzyme. This mutation thus results in a 97% reduction in activity with no significant change in the K_M_ of cytochrome *c* (*t*-test p = 0.2377).

When DCPIP was used as the electron acceptor, the AioB-F108A mutant showed increased activity. WT Aio was previously reported to have a *V*_*max*_ of 4.8 ± 0.03 μmol min^− 1^ mg^− 1^, K_M_ for arsenite of 68.0 ± 4.8 μM and k_cat_ of 8.8 ± 0.7 s^− 1^ (activity values were incorrectly calculated in the original paper due to the use of the wrong extinction coefficient as explained in the methods) [8]. The AioB-F108A mutant had a significantly higher activity than WT Aio with a *V*_*max*_ of 5.8 ± 0.2 μmol min^− 1^ mg^− 1^ (*t*-test p = 0.001). K_M_ for arsenite of 94.6 ± 8.5 and k_cat_ of 10.9 ± 0.4 s^− 1^. To examine the possible contribution of the shift in redox potential of the 2Fe2S cluster on the higher activity, we performed a redox titration of the Rieske from the F108A, followed by EPR. The results demonstrate similar redox potentials at + 205 ± 10 mV (compared to + 225 ± 10 mV in WT). We propose therefore that the higher activity, when DCPIP is used, is most likely due to increased access of DCPIP to the Rieske cluster. This is supported by the crystal structure of the F108A mutant (see below). The improved activity with DCPIP but decreased activity with cytochrome *c* made F108A an interesting mutant to study the nature of the rate-limiting step of catalysis.

Stopped-flow kinetics experiments showed that the F108A mutant had a virtually identical reductive half-reaction to the WT Aio (Fig. 3A), with the reduction of the molybdenum centre (680 nm) too rapid to be observed and the simultaneous reduction of the Fe-S clusters (450 nm) occurring at a rate of 564 ± 46 s^− 1^, which is agreement with the unchanged redox potential of the Rieske cluster. The reduction of cytochrome *c* (551 nm) by Aio and arsenite, on the other hand, was triphasic (Fig. 3B). The first phase appeared to be the reduction of Aio by arsenite which resulted in a decrease in absorbance as opposed to the expected increase seen upon reduction of cytochrome *c*. It was also similar to the value for reduction at 450 nm seen in the previous experiments with a rate of 738 ± 144 s^− 1^. The second phase accounted for 84% of the total absorbance change, with a rate constant of 9.2 ± 0.4 s^− 1^ and corresponding to the reduction of cytochrome *c* by Aio_._ This rate constant is similar to the k_cat_ for the AioB-F108A again implying that reduction of cytochrome *c* is principally the rate-limiting step. The final phase of the reaction had a rate constant of 1.6 ± 0.4 s^− 1^ and again appears to be due to equilibration between Aio and cytochrome *c*. The principal effect of the AioB-F108A substitution is reduced ET to cytochrome *c*.

### 3.4 The crystal structure of the AioB-F108A mutant

**Fig. 4:**
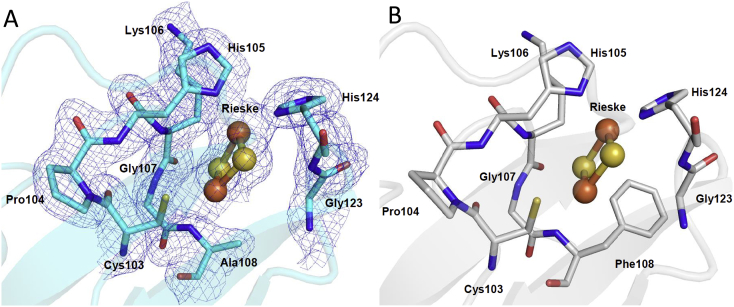
Molecular details of the Rieske cluster of NT-26 AioB. A) Crystal structures of AioB-F108A with the electron density map contoured at 1 σ. B) Crystal structure of the WT AioB.

To structurally characterize AioB-F108A, the mutant was crystallized and the structure determined to 2.2 Å resolution. The obtained model shows that the overall structure is very similar to the WT Aio, with a rmsd of 0.157 Å upon superposition of 843 residues. The refined model fits the electron density map at the mutated site, as observed in Fig. 4. When comparing the structure of WT Aio and AioB-F108A it becomes clear that the side chain of F108 is partially covering the Rieske, acting as a cap that protects the metal cluster from solvent interactions. This effect is not so pronounced for AioB-F108A since the very small side chain of alanine is likely to allow better access of small molecules such as DCPIP to the Fe/S center for electron transfer, which may explain the increased *V*_*max*_ of this mutant with DCPIP.

### 3.5 The AioB-F108A substitution effect on cytochrome *c* binding and affinity

The AioB-F108A substitution significantly reduced the rate of cytochrome *c* reduction in the course of turnover. It could do this either by lowering the affinity of Aio for cytochrome *c* or by affecting the rate of electron transfer, and ITC was thus used to analyse the AioB-F108A/cytochrome *c* affinity. The K_d_ was found to be 7.8 ± 1.7 μM (Fig. 3D). While the difference between the WT Aio and the AioB-F108A mutant is significant (*t*-test p = 0.0067), it is only a 3-fold increase and the data suggest that the AioB-F108A substitution has not dramatically altered cytochrome *c* affinity. The mutation must therefore exert its influence on activity through another means.

A comparison of the binding parameters of cytochrome *c* with WT Aio and AioB-F108A is shown in Table 1. The F108A mutation caused the ∆H to drop by 50% while the ∆S decreased by only 27%. The propensity for endothermic reactions to occur increases as the ∆S becomes more positive and decreases as the ∆H becomes more positive. These results suggest that the presence of F108 has a relatively neutral function in complex formation as both the ΔH and ΔS of cytochrome *c* binding to F108A are smaller compared to WT. It therefore appears that the AioB-F108 is not critical to cytochrome *c* association but instead is important for electron transfer to cytochrome *c*. The AioB-F108 residue thus plays only a small role in the binding process, but once the protein:protein complex forms it is important in bringing the two proteins into the correct orientation for electron transfer and/or in providing an effective electron transfer pathway between the Rieske 2Fe-2S cluster and heme *c* of the cytochrome. The rate of electron transfer exponentially decreases as distance increases meaning a relatively minor change in the complex structure can result in dramatic reductions in rate [51].

The arsenite oxidases appear to show selectivity towards different electron acceptors which appear to coincide with their phylogeny rather than the reduction potential of the electron acceptor [5], [6], [7], [27]. NT-26, a member of the Alphaproteobacteria, can use cytochrome *c* whereas members of the Betaproteobacteria like *A. faecalis* can use some unrelated *c*-type cytochromes and azurin [5], [6], [7]. The main difference between the Rieske subunits is the absence of a disulphide bridge in arsenite oxidases from the Alphaproteobacteria, the cysteines replaced with a glycine and phenylalanine [27]. The presence of the disulphide bridge has been shown to have an significantly smaller effect on the redox potential of the Rieske cluster in Aio (by 35 mV) [8] than in the *bc*_1_ complex (by 54–139 mV) [52], [53], [54]. In the latter, its removal does affect the oxidation of ubiquinol by the *bc*_1_ complex. It has been previously suggested that, in the case of Aio, the presence or absence of the disulphide bridge modulates the electron acceptor selectivity of Aio rather than modulating the redox potential of its Fe-S cluster [7], [8], [9], [10], [11], [12], [13], [14], [15], [16], [17], [18], [19], [20], [21], [22], [23], [24], [25], [26], [27]. Results of this study support this notion as the AioB-F108 appears to be important for electron transfer to cytochrome *c* by bringing the heme of cytochrome *c* closer to the Rieske cluster and/or providing a path for electron transfer.

The following is the supplementary data related to this article.

Table S1 Data collection and refinement statistics (values in parenthesis correspond to the highest resolution shell) Table S1

## Transparency document

Transparency document. Image 2
