# Supplementary material for: Electron transfer through arsenite oxidase: Insights into Rieske interaction with cytochrome c
Source: Biochim Biophys Acta. 2017 Oct;1858(10):865–72. doi: 10.1016/j.bbabio.2017.08.003 (PMC5574378; doi:10.1016/j.bbabio.2017.08.003)
Supplement: Table S1 — Data collection and refinement statistics (values in parenthesis correspond to the highest resolution shell) [file mmc1.docx]

1. **Supplementary Information**

Table S1- Data collection and refinement statistics (values in parenthesis correspond to the highest resolution shell)

| Data Collection (SLS source /PXIII)  Wavelength (Å) | AioB F108A  1.0 |
| --- | --- |
| Resolution (Å) | 49.55-2.20 (2.24-2.20) |
| <I/σ(I)> | 8.2 (1.5) |
| CC_1/2_ | 0.988 (0.590) |
| Total number of observations | 3418983 (157903) |
| Total number of unique obs. | 247069 (12160) |
| R_pim_ (%) | 9.8 (52.4) |
| Multiplicity | 13.8 (13.0) |
| Completeness (%) | 100.0 (100.0) |
| Refinement |  |
| R factor / R _free_ | 18.9 / 22.6 |
| RMSD bond angles (°) | 2.03 |
| RMSD bond lengths (Å) | 0.019 |
| Ramachandran Plot (%)  - residues in most favoured regions  - residues in allowed regions  - residues in disallowed regions | 95.06  4.40  0.54 |
